# Why am I grinding and clenching? Exploration of personality traits, coping strategies, oral parafunctional behaviors, and severe sleep bruxism in a polysomnographic study

**DOI:** 10.3389/fpsyt.2024.1362429

**Published:** 2024-05-22

**Authors:** Tomasz Wieczorek, Anna Jodkowska, Sylwia Orzeszek, Mieszko Wieckiewicz, Monika Michalek-Zrabkowska, Grzegorz Mazur, Joanna Rymaszewska, Joanna Smardz, Anna Wojakowska, Helena Martynowicz

**Affiliations:** ^1^ Department of Psychiatry, Wroclaw Medical University, Wroclaw, Poland; ^2^ Clinical Department of Internal and Occupational Diseases, Hypertension and Clinical Oncology, Wroclaw Medical University, Wroclaw, Poland; ^3^ Department of Experimental Dentistry, Wroclaw Medical University, Wroclaw, Poland; ^4^ Department of Clinical Neuroscience, Wroclaw University of Science and Technology, Wroclaw, Poland

**Keywords:** sleep bruxism, coping strategies, personality, anxiety symptoms, depressive symptoms, oral parafunctional behaviors, polysomnography

## Abstract

**Introduction:**

Causal relationships between psychopathological symptoms, personality traits, coping mechanisms, and sleep bruxism (SB) were studied in the past, giving inconsistent results mostly based on self-assessment evaluations. This polysomnography-based cross-sectional study aimed to explore the relationships between severe SB, personality traits (according to the Big Five model), and coping strategies with objective polysomnographic verification.

**Methodology:**

The study included 66 participants divided into severe SB (SSB) (n=32) and no or mild SB (n=34) groups based on video-polysomnography performed in the sleep laboratory. Questionnaire assessment included the use of the Beck Depression Inventory, Beck Anxiety Inventory, Mini-COPE, International Personality Item Pool Big Five Markers 20-Item version, and Oral Behavior Checklist.

**Results:**

Participants with SSB presented with fewer self-reported anxiety (p=0.008) and depressive (p=0.01) symptoms than the non- or mild-SB groups. The SSB group scored significantly higher in Big Five personal traits such as extraversion (p=0.007), emotional stability (p=0.013), and intellect (p=0.004), while regarding coping strategies, the SSB group was less likely to use negative strategies: self-distraction (p=0.036), denial (p=0.006), venting (p=0.03), behavioral disengagement (p=0.046), and self-blame (p=0.003), and turning to religion (p=0.041). The intensity of oral parafunctional behaviors was comparable in both groups (p=0.054). Emotional stability was a moderate protective factor (p=0.004), and the self-blame strategy was a strong risk factor (p<0.001) for increased oral parafunctional behavior intensity. Phasic activity negatively correlated with anxiety symptom severity (p=0.005), whereas tonic (p=0.122) and mixed (p=0.053) phenotypes did not. SB intensity was a protective factor against anxiety symptoms (p=0.016).

**Conclusion:**

In terms of psychopathology, severe sleep bruxers tend to present less severe anxiety and depressive symptoms, while some of their personality traits (extraversion, emotional stability, and intellect) were more strongly pronounced. SSB is possibly related to the lesser use of the “maladaptive” coping strategies and there were no specific coping strategies preferred by SSB participants, compared to the other group. These observations require further studies, as it should be determined whether SB (especially phasic activity) might be a form of a somatization/functional disorder. Further research should focus on the psychogenic background of oral parafunctional behaviors, which occur more often in less emotionally stable personalities and in people using self-blame coping strategies.

## 1 Introduction

Sleep bruxism (SB) is a masticatory muscle activity during sleep that is characterized as rhythmic (phasic, e.g. tooth grinding) or non-rhythmic (tonic, e.g. tooth clenching) and is not a movement or sleep disorder in otherwise healthy individuals (1). The consequences of SB may include damage to the hard dental tissues, repetitive failures of restorative work/prosthodontic constructions, mechanical wear of the teeth (i.e., attrition), masticatory muscle pain, headache, or limitation of mandibular movements, as well as tooth-grinding sounds that could disrupt the sleep of bed partners (1). According to the present consensus, SB and awake bruxism (AB) are considered two different behaviors as AB is now defined as “a masticatory muscle activity during wakefulness that is characterized by repetitive or sustained tooth contact and/or by bracing or thrusting of the mandible and is not a movement disorder in otherwise healthy individuals (1).

SB is considered a sleep behavior rather than a sleep disorder, and its coexistence with sleep-related disorders and many consequences for oral and overall health are primary and secondary factors to different disorders, such as obstructive sleep apnea, sleep arousals with autonomic activity, sleep-disordered breathing, insomnia, headache, and temporomandibular disorders, or mental dysfunctions such as hypervigilance, increased anxiety/risk of depression, and stress due to life events (2–6). However, SB as a behavior may also have protective properties and positive consequences; it might prevent airway collapse, help restore airway patency in cases of obstructive sleep apnea, and prevent tooth erosion by increasing salivation in patients with gastroesophageal reflux disease (1). Such a complex status of SB indicates the need for a thorough clinical assessment to determine if management is needed and what type of management should be applied. Currently, various treatment approaches are being studied, including oral pharmacotherapy, injectable procedures, oral appliances, biofeedback, counselling, and psychotherapy (7, 8).

Limited literature is available on the correlations between SB and depression, anxiety, coping strategies, and personality traits. Studies performed thus far have provided inconsistent data on the causal relationships between psychopathological symptoms, personality traits, coping mechanisms, and SB (2, 9–13). Some studies mention psychosocial factors as important risk factors; however, the use of serotoninergic agents, mostly selective serotonin reuptake inhibitors (SSRI), is a common risk factor (7, 14, 15). Tobacco smoking has also been reported as a possible risk factor (16). However, some authors have pointed out that AB seems to be more stress-related than SB (15). Additionally, most studies were based on self-assessed SB without polysomnography (PSG) evaluation, which remains a major limitation. Therefore, there is a need to perform focused studies in which SB is objectively assessed using PSG. This study aimed to explore the causalities among severe SB, personality traits (according to the Big Five model), and coping strategies in a relatively homogenous SB group with PSG verification.

## 2 Materials and methods

### 2.1 Participants

Study participants were recruited from patients at the Outpatient Clinic of Temporomandibular Disorders operating at the Department of Experimental Dentistry, Wroclaw Medical University. Patients underwent a comprehensive medical interview and dental inspection, focusing on self-reporting (including bed partner reporting) and signs and symptoms of SB, such as damage to dental hard tissues and oral mucosa, tooth wear, tongue scalloping, and linea alba. All procedures were performed by an experienced dentist. Patients identified with probable SB in accordance with the Third Edition of the International Classification of Sleep Disorders by the American Academy of Sleep Medicine (17) were referred to the Sleep Laboratory at the Department and Clinic of Internal Medicine, Occupational Diseases, Hypertension, and Clinical Oncology at Wroclaw Medical University and underwent a single-night vPSG to confirm SB (1).

The hospitalization was introduced based on purely organizational issues – i.e. in order to perform an overnight polysomnography in the Sleep Lab that is a part of a hospital ward. The study was approved by the Ethics Committee of Wroclaw Medical University (ID KB-794/2019) and was conducted in accordance with the Declaration of Helsinki.

Participants meeting all the following criteria, who also signed an informed consent form, were included in this study: age ≥18 years, suspicion of probable SB (defined as in the consensus by Lobbezoo et al. as “based on self-report plus the inspection part of a clinical examination” (1), which was performed in this study by an experienced dentist), and willingness to participate in the study. The exclusion criteria were as follows: a history of neurological, degenerative, severe mental, or cardiovascular disorders; alcohol and drug addiction; use of antidepressants and drugs that can affect the neuromuscular system; and pregnancy.

After enrolment, the participants were examined using PSG and completed a battery of questionnaires.

### 2.2 Polysomnography evaluation

All patients underwent overnight video-PSG using Nox-A1 (Nox Medical) at the Sleep Laboratory of the Department of Internal Medicine, Occupational Diseases, Hypertension and Clinical Oncology at Wroclaw Medical University. Based on the standard criteria for sleep scoring recommended by the American Academy of Sleep Medicine (AASM), manual assessment of the registered data was performed on a 30 s epoch basis. The following registered parameters were included: total sleep time (TST), sleep latency (SL), rapid eye movement (REM), REM latency, sleep efficiency (SE), duration of wake episodes after sleep onset (WASO), percentage of non-REM sleep stages 1–3 (NREM1–3) and percentage of REM sleep. Abnormal respiratory events were scored according to the standard criteria of the AASM Task Force (18), and the following parameters were measured: apnea–hypopnea index (AHI), average blood oxygen saturation (SpO2), minimal SpO2, time with SpO2 < 90% and average desaturation drop. Apnea is defined as the absence of airflow for ≥10 s. Hypopnea is defined as the reduction in the amplitude of breathing by ≥30% for ≥10 s with a ≥3% decline in the blood oxygen saturation leading to arousal from sleep. AHI is defined as the number of apnea or hypopnea episodes per one hour of sleep.

Definite SB was evaluated through bilateral masseter electromyography (EMG) and audio-video recordings. The following indices were examined: the bruxism episodes index (BEI), phasic bruxism (characterized by more than three cyclic phasic EMG increases lasting 0.25–2 s), tonic bruxism (episodes lasting >2 s), and mixed bruxism (a combination of both types of episodes mentioned). SB episodes were scored after a minimum of 3 s of stable electromyography, and when the activity was at least twice the amplitude of the background electromyography (1, 17). SB was categorized based on the frequency of bruxism episodes per hour of sleep (BEI) as non-SB (BEI < 2), mild to moderate SB (BEI 2–4), or severe SB (BEI > 4) (1, 19).

### 2.3 Psychometric tools

- International Personality Item Pool Big Five Markers 20-Item version (IPIP-BFM-20), prepared and published by Topolewska et al. (20) based on the Polish IPIP-BFM-50 questionnaire by Strus et al. (21) and shortened guidelines by Donnellan et al. (22). This 20-item self-assessment questionnaire is used to measure the Big Five personality traits: extraversion, agreeableness, conscientiousness, emotional stability, and intellect. Items are rated on a 1–5-point Likert scale, and each of the measured traits is assessed with four items; therefore, the score for each trait is placed within the range of 4–20 points.
- The Mini-COPE was developed by Carver et al. (23, 24). This tool has been designed and validated for use with adults. A questionnaire is used to assess ways of coping with stress. It is intended mainly for research purposes but can also be used in practice, screening, and prophylactic tests to assess the effectiveness of therapeutic effects. It is a 28-item self-assessment questionnaire that evaluates preferred coping strategies (i.e., active coping, planning, positive reframing, acceptance, sense of humor, turning to religion, seeking emotional support, seeking instrumental support, self-distraction, denial, venting, substance use, behavioral disengagement, and self-blame). Each of these strategies is assessed with 2 item scores, giving a total of 28 items. A 0–3-point Likert scale is used for each item.
- The Beck Depression Inventory-II (BDI-II tool was created by Beck et al. (25). This worldwide self-assessment tool consisting of 21 items is used to measure depressive symptoms for research and clinical purposes. Each item is rated on a 0–3-point Likert scale. Scores of 0–11 are suggestive for no depression, 12–19 for mild depression, 20–25 for moderate depression, and 26–63 for severe depression.
- The Beck Anxiety Inventory (BAI) is another tool developed by Beck et al. (26). It is a self-assessment tool designed to evaluate the clinical symptoms of anxiety, focusing mostly on bodily and somatic symptoms. The inventory consists of 21 items rated on a 0–3-point Likert scale. The sum of all items is counted. A score of 7 points or below is considered a minimal level of anxiety, 8–15 points means a mild level, 16–25 points indicate moderate anxiety severity, and 26 or more points indicates a severe anxiety level possibly related to anxiety disorders (26).
- The Oral Behavior Checklist (OBC), created by Markiewicz et al. (27), is a tool for the self-assessment of the number and frequency of parafunctional oral behaviors, which was verified and validated in electromyographic studies (28). It has been proposed as part of the diagnostic process for temporomandibular disorders (TMDs) (29). It consists of 21 items that assess different oral behaviors. Each item is rated on a 0–4-point Likert scale, yielding a total sum ranging from 0 to 84 points. A score higher than 24 points is considered a risk factor for TMDs (29).

### 2.4 Statistical analysis

Statistical analysis was performed using the “Statistica 13” software by TIBCO Software (Poland). Qualitative variables (gender) were analyzed with the use of Chi-square test. The Shapiro–Wilk test and histogram visual analysis were performed to test the normal distribution of the data. Student’s t-test for parametric data and the Mann–Whitney U-test for nonparametric data were performed to test the significance of differences in the mean values between the two groups. Correlation analysis was performed using Spearman’s correlation rank test. The significantly correlated variables were later verified as possible risk factors or protective factors using univariate linear regression models. Significant factors were subsequently introduced into multifactorial stepwise regression analyses to determine whether they remained significant in the multivariate models. Statistical significance was set at p < 0.05 in the case of all used statistical tools. For the purposes of the study (focus on severe SB) and statistical analysis, study participants were divided into two groups: SSB (>4 episodes of bruxism per hour of sleep) and NSB&MSB (none or up to 4 episodes per hour of sleep).

## 3 Results

In the first stage, 82 participants were recruited; however, after PSG evaluation, 16 participants were excluded from the study because of a diagnosis of obstructive apnea syndrome (based on AHI > 5). Finally, 66 participants were enrolled in the study (mean age, 34.3 ± 9.68 years; 50 females and 16 males). Based on the bruxism episode index (BEI) measured in PSG evaluation, the group was further divided into subgroups: no sleep bruxism (NSB, BEI<2) n=17; 15 females and 2 males), mild sleep bruxism (MSB, 2≤BEI<4, n=17; 14 females and 3 males), and severe sleep bruxism (SSB, BEI≥4, n=32; 21 females and 11 males). Because of the relatively low number of participants in the first two subgroups and one of the basic premises of this study (i.e. to focus on severe bruxism assessment), most of the analyses were performed with them combined into the NSB&MSB subgroup (n=34; 29 females and 5 males). What is more, in the study by Lavigne et al. a cut-off score of BEI=4 was recommended for the diagnosis of clinically significant SB (19). Several other studies have also introduced this methodology of participant division (30, 31). In terms of PSG parameters, significant differences between subgroups were measured for all SB parameters (BEI, Phasic BEI, Tonic BEI, Mixed BEI) and N1 sleep percentage (which was significantly higher in the SSB subgroup). The Chi-square test revealed no significant difference in terms of gender structure between both subgroups (p=0.062). Details of age, height, weight, BMI, and PSG parameters are shown in **Table 1**.

**Table 1:** Basic and PSG data in the whole group and subgroups.

	Whole group (n=66)		NSB&MSB (n=34)		SSB (n=32)		Intergroup difference (p value)
	Mean (SD)	Median (IQR)	Mean (SD)	Median (IQR)	Mean (SD)	Median (IQR)	
Age [year]	34.30 (9.68)	33.00 (13.0)	35.26 (9.99)	33.00 (10.0)	33.28 (9.38)	32.00 (13.0)	0.41
Height [cm]	169.68 (9.62)	170.00 (12.0)	168.94 (8.60)	168.50 (11.0)	170.47 (10.68)	170.50 (14.5)	0.52
Weight [kg]	70.03 (16.44)	67.00 (18.0)	71.53 (17.47)	67.00 (16.0)	68.44 (15.38)	66.50 (21.0)	0.62
BMI [kg/m2]	23.96 (3.75)	23.30 (5.1)	24.56 (3.92)	23.55 (4.7)	23.33 (3.51)	23.15 (5.35)	0.186
BEI [episodes/min]	4.43 (3.16)	3.55 (4.3)	2.04 (0.90)	2.05 (1.1)	6.97 (2.68)	6.35 (3.3)	<0.001*
Phasic BEI [episodes/h]	2.63 (2.61)	1.90 (2.9)	1.01 (0.78)	0.90 (1.2)	4.35 (2.77)	3.85 (2.75)	<0.001*
Tonic BEI [episodes/h]	1.06 (0.75)	0.90 (1.0)	0.65 (0.38)	0.55 (0.5)	1.50 (0.79)	1.30 (0.95)	<0.001*
Mixed BEI [episodes/h]	0.77 (0.61)	0.60 (0.7)	0.39 (2.45)	0.40 (0.4)	1.18 (0.61)	1.15 (1.05)	<0.001*
AHI [episodes/h]	2.12 (1.2)	2.05 (1.4)	2.01 (1.10)	2.05 (1.4)	2.25 (1.3)	2.05 (1.75)	0.80
TST [min]	443.33 (59.79)	450.45 (85.00)	449.60 (58.33)	461.25 (86.0)	436.66 (61.52)	446.95 (70.75)	0.37
SL [min]	21.33 (27.4)	12.85 (18.6)	20.94 (33.45)	11.95 (15.5)	21.74 (19.56)	15.75 (27.9)	0.51
REM Latency [min]	88.60 (62.01)	72.75 (40.5)	80.18 (41.45)	72.50 (40.0)	97.56 (77.94)	78.00 (42.65)	0.75
WASO [min]	37.20 (32.7)	25.85 (36.5)	40.80 (30.95)	27.60 (43.5)	33.38 (34.55)	25.30 (27.45)	0.28
SE [%]	87.19 (9.85)	89.60 (11.8)	86.50 (10.04)	89.00 (14.7)	87.92 (9.75)	90.10 (9.3)	0.48
NREM 1 [%]	3.98 (2.85)	3.00 (3.0)	3.12 (2.35)	2.55 (2.10)	4.90 (3.07)	4.20 (3.8)	0.004*
NREM 2 [%]	46.64 (9.81)	46.25 (9.4)	46.10 (6.22)	45.35 (7.80)	47.21 (12.65)	47.40 (14.95)	0.56
NREM 3 [%]	24.47 (6.32)	23.00 (8.8)	24.45 (6.29)	23.25 (9.90)	24.48 (6.45)	23.00 (7.75)	0.98
REM [%]	24.90 (7.59)	26.15 (9.4)	26.32 (5.97)	26.35 (5.90)	23.39 (8.84)	25.20 (11.3)	0.27
Average SpO2 [%]	94.94 (0.98)	95.10 (1.2)	94.76 (1.10)	94.90 (1.2)	95.13 (0.81)	95.10 (1.1)	0.28
Min SpO2 [%]	85.45 (8.42)	88.00 (11.0)	87.50 (5.14)	88.50 (7.0)	83.19 (10.60)	85.00 (13.00)	0.19
SpO2 <90% [%]	2.1 (4.32)	0.10 (2.2)	1.49 (2.96)	0.10 (1.2)	2.74 (5.38)	0.20 (3.5)	0.57
Average Desaturation Drop [%]	3.29 (0.61)	3.20 (0.5)	3.28 (0.44)	3.25 (0.5)	3.30 (0.76)	3.20 (0.5)	0.70

Statistically significant differences are marked in bold and with an asterisk [*]. AHI, Apnea/Hypopnea Index; BEI, Bruxism Episode Index; BMI, Body Mass Index; MSB, Mild Sleep Bruxism; NREM, Non-Rapid Eye Movement; NSB, No Sleep Bruxism; REM, Rapid Eye Movement; SE, Sleep Efficiency; SL, Sleep Latency; SpO2, Blood Oxygen Saturation; SSB, Severe Sleep Bruxism; TST, Total Sleep Time; WASO, Wake After Sleep Onset.

### 3.1 The Big Five personality traits, coping strategies, subjective depressive and anxiety symptoms, and oral parafunctional behaviors

The Big Five personality trait scores measured using the IPIP BFM-20 tool were compared between the subgroups. Differences were observed in extraversion, emotional stability, and intellect; all three traits were significantly more expressed in the SSB subgroup. Regarding coping strategies, the SSB group was less likely to use religion, self-distraction, denial, venting, behavioral disengagement, and self-blame. A high risk of oral parafunctional behaviors (measured with OBC) was present in 20 SSB (66.7%) and 25 NSB&MSB (73.5%) subgroup participants. The OBC scores did not differ significantly between subgroups. Subjective depressive and anxiety symptoms (measured using the BDI and BAI, respectively) were significantly more pronounced in the NSB&MSB subgroup. Detailed information is presented in **Table 2**.

**Table 2:** IPIP BFM-20, Mini-COPE, and OBC scores in the whole group and subgroups.

	Whole group (N=66)		NSB&MSB (n=34)		SSB (n=32)		Intergroup difference (p value)
	Mean (SD)	Median (IQR)	Mean (SD)	Median (IQR)	Mean (SD)	Median (IQR)	
**IPIP extraversion**	**12.83 (3.94)**	**13.00 (6.0)**	**11.59 (4.12)**	**11.50 (7.0)**	**14.19 (3.28)**	**14.00 (4.0)**	0.007*
IPIP agreeableness	15.57 (2.71)	16.00 (4.0)	15.65 (2.56)	15.50 (5.0)	15.48 (2.91)	16.00 (4.0)	0.95
IPIP conscientiousness	15.06 (2.97)	15.00 (3.0)	14.65 (3.14)	15.00 (4.0)	15.51 (2.76)	16.00 (4.0)	0.24
**IPIP emotional stability**	**10.85 (3.43)**	**10.00 (4.0)**	**9.85 (3.4)**	**10.00 (5.0)**	**11.94 (3.16)**	**12.00 (5.0)**	0.013*
**IPIP intellect**	**14.69 (2.83)**	**15.00 (3.0)**	**13.74 (3.04)**	**13.50 (4.0)**	**15.74 (2.19)**	**16.00 (4.0)**	0.004*
MC active coping	2.46 (0.6)	2.50 (1.0)	2.41 (0.54)	2.25 (1.0)	2.52 (0.66)	2.75 (1.0)	0.27
MC planning	2.4 (0.56)	2.50 (1.0)	2.41 (0.50)	2.50 (1.0)	2.38 (0.64)	2.50 (1.0)	0.93
MC positive reframing	1.70 (0.72)	2.00 (1.0)	1.62 (0.76)	1.50 (1.0)	1.80 (0.68)	2.00 (0.5)	0.32
MC acceptance	1.96 (0.70)	2.00 (1.0)	2.01 (0.70)	2.00 (1.0)	1.90 (0.71)	2.00 (1.0)	0.46
MC sense of humor	0.82 (0.54)	0.75 (0.5)	0.85 (0.50)	1.00 (0.5)	0.78 (0.58)	0.50 (0.5)	0.38
**MC turning to religion**	**0.75 (0.97)**	**0.00 (1.5)**	**0.99 (1.03)**	**0.75 (2.0)**	**0.48 (0.84)**	**0.00 (1.0)**	0.041*
MC seeking emotional support	2.04 (0.80)	2.00 (1.0)	2.06 (0.62)	2.00 (0.5)	2.02 (0.97)	2.00 (1.5)	0.76
MC seeking instrumental support	1.98 (0.75)	2.00 (0.75)	2.01 (0.70)	2.00 (0.5)	1.95 (0.81)	2.00 (1.0)	0.60
**MC self-distraction**	**1.59 (0.78)**	**1.50 (1.0)**	**1.79 (0.73))**	**2.00 (0.5)**	**1.37 (0.79)**	**1.50 (1.5)**	0.036*
**MC denial**	**0.59 (0.71)**	**0.50 (1.0)**	**0.78 (0.69)**	**1.00 (1.0)**	**0.38 (0.69)**	**0.00 (0.5)**	0.005*
**MC venting**	**1.41 (0.71)**	**1.50 (1.0)**	**1.59 (0.60)**	**1.50 (1.0)**	**1.20 (0.77)**	**1.50 (1.0)**	0.030*
MC substance use	0.39 (0.60)	0.00 (1.0)	0.41 (0.69)	0.00 (0.5)	0.37 (0.49)	0.00 (1.0)	0.90
**MC behavioral disengagement**	**0.63 (0.54)**	**0.50 (1.0)**	**0.75 (0.53)**	**1.00 (0.5)**	**0.50 (0.53)**	**0.50 (1.0)**	0.046*
**MC self-blame**	**1.24 (0.83)**	**1.00 (0.75)**	**1.53 (0.79)**	**1.50 (1.0)**	**0.92 (0.76)**	**1.00 (1.5)**	0.003*
OBC	35.42 (11.97)	36.00 (18.0)	38.41 (12.08)	39.00 (15.00)	32.32 (11.24)	33.50 (17.5)	0.054
**BDI**	**9.62 (8.04)**	**8.00 (12.0)**	**11.91 (7.83)**	**11.00 (12.0)**	**7.10 (7.62)**	**4.00 (9.0)**	0.01*
**BAI**	**16.78 (12.93)**	**15.00 (17.00)**	**21.15 (13.9)**	**19.00 (16.00)**	**12.13 (10.09)**	**10.00 (16.00)**	0.008*

Statistically significant differences are marked in bold and with an asterisk [*]. BAI, Beck Anxiety Inventory; BDI, Beck Depression Inventory; IPIP, International Personality Item Pool; MC, Mini-COPE; MSB, Mild Sleep Bruxism; NSB, No Sleep Bruxism; OBC, Oral Behavior Checklist; SSB, Severe Sleep Bruxism.

### 3.2 Correlations of Big Five personality traits, coping strategies, and SB parameters

Spearman’s rank correlation coefficients were calculated for all pairs of the Big Five personality traits and measured SB parameters (including BEI and indices for specific SB activity phenotypes). Extraversion, emotional stability, and intellect were positively and weakly (though significantly) correlated with the BEI. Emotional stability was also positively and weakly correlated with the phasic BEI and with the mixed BEI. Intellect was also positively and weakly correlated with the tonic BEI. Details of the measured correlations are listed in **Table 3**.

**Table 3:** Spearman correlations of Big Five personality traits and Sleep Bruxism Phenotype activity.

	R Spearman	t(N-2)	p
**IPIP extraversion & BEI**	**0.254**	**2.086**	0.041*
IPIP extraversion & Phasic BEI	0.203	1.647	0.104
IPIP extraversion & Tonic BEI	0.155	1.246	0.217
IPIP extraversion & Mixed BEI	0.244	1.994	0.050
IPIP agreeableness & BEI	-0.051	-0.409	0.684
IPIP agreeableness & Phasic BEI	-0.132	-1.056	0.295
IPIP agreeableness & Tonic BEI	0.155	1.244	0.218
IPIP agreeableness & Mixed BEI	-0.072	-0.573	0.569
IPIP conscientiousness & BEI	0.148	1.185	0.241
IPIP conscientiousness & Phasic BEI	0.150	1.204	0.233
IPIP conscientiousness & Tonic BEI	0.038	0.299	0.766
IPIP conscientiousness & Mixed BEI	0.093	0.745	0.459
**IPIP emotional stability & BEI**	**0.277**	**2.287**	0.026*
**IPIP emotional stability & Phasic BEI**	**0.248**	**2.030**	0.047*
IPIP emotional stability & Tonic BEI	0.194	1.567	0.122
**IPIP emotional stability & Mixed BEI**	**0.302**	**2.510**	0.015*
**IPIP intellect & BEI**	**0.256**	**2.104**	0.039*
IPIP intellect & Phasic BEI	0.130	1.043	0.301
**IPIP intellect & Tonic BEI**	**0.302**	**2.510**	0.015*
IPIP intellect & Mixed BEI	0.193	1.560	0.124

Statistically significant correlations are marked in bold and with an asterisk [*]. BEI, Bruxism Episode Index; IPIP, International Personality Item Pool.

Spearman’s rank correlation coefficients were calculated for all pairs of coping strategies (measured using the Mini-COPE) and SB parameters (including the BEI and indices for specific SB activity phenotypes). Turning to religion, denial, and behavioral disengagement were negatively and weakly (although significantly) correlated with BEI, whereas venting and self-blame were moderately and negatively correlated with BEI. Self-blame was also negatively and weakly correlated with phasic BEI and mixed BEI, and negatively and moderately with Tonic BEI. A relatively strong negative correlation was observed between the venting and mixed BEI, while denial was weakly and negatively correlated with mixed BEI. Turning to religion was correlated weakly and negatively with the phasic BEI. Details of the measured correlations are listed in **Table 4**.

**Table 4:** Spearman correlations of coping strategies and sleep bruxism phenotype activity.

	R Spearman	t(N-2)	p
MC active coping & BEI	0,097	0,768	0,446
MC active coping & Phasic BEI	0,067	0,531	0,598
MC active coping & Tonic BEI	0,173	1,385	0,171
MC active coping & Mixed BEI	0,003	0,024	0,981
MC planning & BEI	-0,027	-0,210	0,834
MC planning & Phasic BEI	-0,077	-0,612	0,543
MC planning & Tonic BEI	0,109	0,864	0,391
MC planning & Mixed BEI	0,011	0,089	0,929
MC positive reframing & BEI	0,049	0,387	0,700
MC positive reframing & Phasic BEI	0,021	0,168	0,867
MC positive reframing & Tonic BEI	0,126	0,999	0,321
MC positive reframing & Mixed BEI	0,015	0,118	0,907
MC acceptance & BEI	-0,139	-1,102	0,275
MC acceptance & Phasic BEI	-0,116	-0,916	0,363
MC acceptance & Tonic BEI	-0,107	-0,846	0,401
MC acceptance & Mixed BEI	-0,125	-0,993	0,324
MC sense of humor & BEI	-0,061	-0,482	0,632
MC sense of humor & Phasic BEI	-0,077	-0,609	0,545
MC sense of humor & Tonic BEI	-0,135	-1,075	0,287
MC sense of humor & Mixed BEI	-0,054	-0,428	0,670
**MC turning to religion & BEI**	**-0,260**	**-2,118**	0,038*
**MC turning to religion & Phasic BEI**	**-0,339**	**-2,840**	0,006*
MC turning to religion & Tonic BEI	-0,195	-1,568	0,122
MC turning to religion & Mixed BEI	-0,119	-0,941	0,350
MC seeking emotional support & BEI	0,011	0,084	0,933
MC seeking emotional support & Phasic BEI	-0,024	-0,189	0,851
MC seeking emotional support & Tonic BEI	0,152	1,214	0,229
MC seeking emotional support & Mixed BEI	0,078	0,616	0,540
MC seeking instrumental support & BEI	-0,136	-1,081	0,284
MC seeking instrumental support & Phasic BEI	-0,205	-1,653	0,103
MC seeking instrumental support & Tonic BEI	0,027	0,214	0,831
MC seeking instrumental support & Mixed BEI	-0,004	-0,028	0,978
MC self-distraction & BEI	-0,147	-1,173	0,245
MC self-distraction & Phasic BEI	-0,100	-0,790	0,432
MC self-distraction & Tonic BEI	-0,243	-1,971	0,053
MC self-distraction & Mixed BEI	-0,184	-1,476	0,145
**MC denial & BEI**	**-0,254**	**-2,068**	0,043*
MC denial & Phasic BEI	-0,205	-1,647	0,105
MC denial & Tonic BEI	-0,228	-1,844	0,070
**MC denial & Mixed BEI**	**-0,249**	**-2,022**	0,048*
**MC venting & BEI**	**-0,330**	**-2,754**	0,008*
MC venting & Phasic BEI	-0,237	-1,918	0,060
MC venting & Tonic BEI	-0,238	-1,927	0,059
**MC venting & Mixed BEI**	**-0,421**	**-3,656**	0,001*
MC substance use & BEI	0,057	0,451	0,654
MC substance use & Phasic BEI	0,093	0,732	0,467
MC substance use & Tonic BEI	0,051	0,402	0,689
MC substance use & Mixed BEI	-0,047	-0,373	0,710
**MC behavioral disengagement & BEI**	**-0,251**	**-2,041**	0,046*
MC behavioral disengagement & Phasic BEI	-0,236	-1,913	0,060
MC behavioral disengagement & Tonic BEI	-0,116	-0,923	0,359
MC behavioral disengagement & Mixed BEI	-0,157	-1,253	0,215
**MC self-blame & BEI**	**-0,320**	**-2,661**	0,010*
**MC self-blame & Phasic BEI**	**-0,250**	**-2,030**	0,047*
**MC self-blame & Tonic BEI**	**-0,386**	**-3,292**	0,002*
**MC self-blame & Mixed BEI**	**-0,262**	**-2,138**	0,036*

Statistically significant correlations are marked in bold and with an asterisk [*]. BEI, Bruxism Episode Index; MC, Mini-COPE.

Single-factor regression analysis models were created based on the results of the correlation analysis. None of the Big Five personality traits were significant predictors of either BEI or different SB activity phenotypes. Multifactorial stepwise regression models with backward elimination were created for BEI, Phasic BEI and Mixed BEI, including the coping strategies that were significant predictors in the single-factor regression analyses. In the final model, only the behavioral disengagement strategy was a relatively weak protective (negative) factor for BEI. No significant factors were found in case of the Phasic activity. Venting turned out to be a possible weakly protective (negative) factor for Mixed BEI. As self-blame strategy was an only significantly correlated factor in case of Tonic BEI, only a single factor analysis for this parameter was performed and it revealed that self-blame strategy is a moderately protective (negative) factor for Tonic activity. The details are presented in **Table 5**.

**Table 5:** Multifactorial stepwise regression results including coping strategies that were predictors of BEI or sleep bruxism phenotype activity score in single factor regression models.

SB parameter		MS	F	p	β	R2
BEI	Intercept	753.806	80.739	<0.001	-	0.072
	MC turning to religion	Ns	Ns	Ns	Ns	
	MC denial	Ns	Ns	Ns	Ns	
	MC venting	Ns	Ns	Ns	Ns	
	**MC behavioral disengagement**	**45.230**	**4.845**	0.031*	**-0.269**	
	MC self-blame	Ns	Ns	Ns	Ns	
Phasic BEI	Intercept	416.670	62.524	<0.001	-	0.00
	MC turning to religion	Ns	Ns	NS	Ns	
	MC self-blame	Ns	Ns	Ns	Ns	
Mixed BEI	Intercept	15.471	46.049	<0.001	-	0.072
	MC denial	Ns	Ns	Ns	Ns	
	**MC venting**	**1.990**	**5.923**	0.018*	**-0.295**	
	MC self-blame	Ns	Ns	Ns	Ns	
Tonic BEI^a^	Intercept	43.755	88.564	<0,001	-	0.13
	**MC self-blame**	**5.139**	**10.401**	0,002*	**-0.379**	

Statistically significant p value is marked in bold and with an asterisk [*]. ^a^ – in case of Tonic activity, only one factor was correlated significantly, so the scores presented in the table for Tonic activity analysis base on the single factor regression model. BEI, Bruxism Episode Index; MC, Mini-COPE; Ns, not significant.

### 3.3 Correlations of oral parafunctional behaviors and SB parameters

The OBC score was weakly and negatively correlated with BEI. No significant correlations were found for specific SB activity phenotypes. The details are listed in **Table 6**.

**Table 6:** Spearman correlations of Oral Behavior Checklist score and sleep bruxism phenotype activity.

	R Spearman	t(N-2)	p
**Oral Behavior Checklist & BEI**	**-0.271**	**-2.088**	0.041*
Oral Behavior Checklist & Phasic BEI	-0.257	-1.973	0.054
Oral Behavior Checklist & Tonic BEI	-0.232	-1.769	0.082
Oral Behavior Checklist & Mixed BEI	-0.183	-1.377	0.174

Significant correlation is marked in bold and with an asterisk [*]. BEI, Bruxism Episode Index.

### 3.4 Correlations of Big Five personality traits, coping strategies and oral parafunctional behaviors

Spearman’s rank correlation coefficients were calculated for all pairs of the Big Five personality traits (IPIP BFM-20) or coping strategies (Mini-COPE), and oral parafunctional behaviors (OBC). Relatively strong correlations were found for the emotional stability trait (negative correlation) and self-distraction and self-blame strategies (positive correlations). Substance use and venting were moderately and positively correlated with OBC scores, whereas denial strategy was weakly and positively correlated. The details are presented in **Table 7**.

**Table 7:** Spearman correlations of Oral Behavior Checklist, Big Five personality traits, and coping strategies.

	R Spearman	t(N-2)	p
IPIP extraversion & OBC	-0.064	-0.473	0.638
IPIP agreeableness & OBC	0.104	0.770	0.444
IPIP conscientiousness & OBC	-0.076	-0.564	0.575
**IPIP emotional stability & OBC**	**-0.516**	**-4.424**	<0.001*
IPIP intellect & OBC	0.066	0.485	0.629
MC active coping & OBC	-0.083	-0.605	0.548
MC planning & OBC	0.202	1.503	0.139
MC positive reframing & OBC	-0.131	-0.961	0.341
MC acceptance & OBC	-0.084	-0.616	0.541
MC sense of humor & OBC	0.251	1.886	0.065
MC turning to religion & OBC	0.061	0.441	0.661
MC seeking emotional support & OBC	0.095	0.691	0.492
MC seeking instrumental support & OBC	0.164	1.207	0.233
**MC self-distraction & OBC**	**0.516**	**4.383**	<0.001*
**MC denial & OBC**	**0.291**	**2.211**	0.031*
**MC venting & OBC**	**0.332**	**2.565**	0.013*
**MC substance use & OBC**	**0.377**	**2.965**	0.005*
MC behavioral disengagement & OBC	-0.021	-0.151	0.881
**MC self-blame & OBC**	**0.616**	**5.693**	<0.001*

Significant correlations are marked in bold and with an asterisk [*]. IPIP, International Personality Item Pool; MC, Mini-COPE; OBC, Oral Behavior Checklist.

Based on the results of the correlation analysis, a multifactorial stepwise regression model with backward elimination was created that included variables that were significantly correlated with OBC. In the model, emotional stability was a relatively moderate protective (negative) factor, and the self-blame strategy was a relatively strong risk (positive) factor for increased OBC scores. The details are listed in **Table 8**.

**Table 8:** Multifactorial stepwise regression results including coping strategies that were correlated with Oral Behavior Checklist as single factors.

	MS	F	p	β	R2
Intercept	4317.062	54.170	<0.001		0.452
**IPIP emotional stability**	**745.708**	**9.357**	0.004*	**-0.332**	
MC self-distraction	Ns	Ns	Ns	Ns	
MC denial	Ns	Ns	Ns	Ns	
MC venting	Ns	Ns	Ns	Ns	
MC substance use	Ns	Ns	Ns	Ns	
**MC self-blame**	**1668.724**	**20.939**	<0.001*	**0.496**	

Statistically significant p values are marked in bold and with an asterisk [*]. IPIP, International Personality Item Pool; MC, Mini-COPE; Ns, not significant.

### 3.5 Correlations of subjective depressive and anxiety symptoms with oral parafunctional behaviors and SB activity

Spearman’s rank correlation coefficients were calculated for all pairs of subjective depressive (BDI) and anxiety (BAI) symptoms, BEI, and oral parafunctional behaviors (OBC). Strong positive correlations were found between OBC scores and both depressive and anxiety symptoms, whereas moderately negative correlations were found for both anxiety and depressive symptoms and BEI. In the case of SB phenotypes, Tonic and Phasic BEI showed significant, though very weak, negative correlations with the BDI score. The BAI score correlated moderately in a negative manner with the Phasic BEI but not with Tonic BEI. The details are presented in **Table 9**.

**Table 9:** Spearman correlations of depressive/anxiety symptoms, Oral Behavior Checklist Score, and sleep bruxism phenotype activity.

	R Spearman	t(N-2)	p
**BDI & BEI**	**-0.306**	**-2.534**	0.014*
**BDI & OBC**	**0.550**	**4.790**	<0.001*
**BAI & BEI**	**-0.357**	**-2.985**	0.004*
**BAI & OBC**	**0.656**	**6.262**	<0.001*
**BDI & Phasic BEI**	**-0.267**	**-2.182**	0.033*
**BDI & Tonic BEI**	**-0.265**	**-2.161**	0.035*
BDI & Mixed BEI	-0.215	-1.735	0.088
**BAI & Phasic BEI**	**-0.351**	**-2.925**	0.005*
BAI & Tonic BEI	-0.197	-1.567	0.122
BAI & Mixed BEI	-0.245	-1.975	0.053

Significant correlations are marked in bold and with an asterisk [*]. BAI, Beck Anxiety Inventory; BDI, Beck Depression Inventory; BEI, Bruxism Episode Index; OBC, Oral Behavior Checklist.

Based on the results of the correlation analysis, two univariate regression models were created, including variables that were significantly correlated with the BEI. SB activity was a possible protective (negative) factor against subjective anxiety symptoms, whereas this effect was not observed for subjective depressive symptoms. Details are presented in **Tables 10**, **11**.

**Table 10:** Univariate linear regression model with BAI score set as dependent variable.

	MS	F	p	β	R2
Intercept	9464.343	60.555	<0.001	-	0.075
**BEI**	**952.030**	**6.091**	0.016*	**-0.30**	

Statistically significant factor is marked in bold and with an asterisk [*]. BEI, Bruxism Episode Index.

**Table 11:** Univariate linear regression model with BDI score set as dependent variable.

	MS	F	p	β	R2
Intercept	2694.859	43.525	<0.001	-	0.027
BEI	170.994	2.762	0.102	-0.21	

BEI, Bruxism Episode Index.

### 3.6 Correlations of apnea/hypopnea index and SB activity

Spearman’s rank correlation coefficients were calculated for all pairs of AHI and measured SB parameters (including BEI and indices for specific SB activity phenotypes). No significant correlations were found between the AHI and BEI or the different SB phenotypes. The details are listed in **Table 12**.

**Table 12:** Spearman correlations of Apnea/Hypopnea Index and sleep bruxism phenotype activity.

	R Spearman	t(N-2)	p
AHI & BEI	0,009018	0,072146	0,942711
AHI & Phasic BEI	-0,003386	-0,027091	0,978472
AHI & Tonic BEI	0,052415	0,419898	0,675966
AHI & Mixed BEI	0,074219	0,595391	0,553681

AHI, Apnea/Hypopnea Index; BEI, Bruxism Episode Index.

## 4 Discussion

There have been inconsistent and limited PSG-based data available in the literature focusing on the analysis of correlations between SB and depression, anxiety, coping strategies, and personality traits. Thus, the causative-consequence relationship remains unclear (2, 9–13).

The most important finding of our study was that participants with PSG-confirmed SSB clearly presented less self-reported somatic anxiety and depressive symptoms (measured with BAI and BDI) compared with the no or mild SB group. Additionally, we observed that in SSB individuals, scores in the Big Five personal traits, such as extraversion, emotional stability, and intellect, were significantly higher than in the non-SB/mild-SB group. Regarding coping strategies, the SSB group was less likely to use negative strategies, such as self-distraction, denial, venting, behavioral disengagement, and self-blame, as well as turning to religion. The present observation is in agreement with a previous PSG-based study by Smardz et al., which showed a lack of significant depressive symptoms (measured using the BDI) in bruxers compared with those in non-bruxers (12).

In general view, stress and anxiety were considered to be significant components in the pathogenesis of bruxism and the overall opinion exists that they could be correlated with sleep bruxism (2, 3, 9–11, 32). Winocur et al. observed that one of the variables affecting the occurrence of sleep bruxism in Israeli adolescents was anxiety and stress together with temporomandibular symptoms (9). In a study by Itani et al., decreased positive and depressive feelings were found to be factors associated with sleep-related bruxism in Japanese adolescents (10). Schneider and Schaefer reported that self-reported sleep bruxers used fewer positive coping strategies, indicating a deficit in functional coping strategies (13). However, the findings did not indicate a causal association. These observations are clearly different from those of our PSG study, as in our observations the SSB subgroup (compared to the no or mild SB subgroup) was less likely to use strategies mostly considered as negative (self-distraction, denial, venting, behavioral disengagement, and self-blame) and there were no significant correlations (either positive or negative) in case of positive strategies. Additionally, in our findings SSB was correlated with less severe self-reported anxiety and depressive symptoms.

One of the possible explanations for the fact that sleep bruxers showed fewer negative coping strategies in our study is that SB should be considered a sleep behavior rather than a sleep disorder, which is consistent with the current definition and consensus (1). In an older neurobiological study, SB was proposed as an extreme manifestation of rhythmic masticatory muscle activity (RMMA) occurring during sleep in most healthy participants, as RMMA was observed in 60% of normal sleepers in the absence of grinding sounds (33). The observation in our study that the oral parafunctional behavior (OBC) score is negatively correlated with SB (BEI) supports this approach and is in agreement with what has been previously shown in PSG-based and other studies—that TMD and SB are not the same (6, 34, 35). In our study it cannot be excluded that SSB participants could have been more likely to take part in the study, because they were expressing less maladaptive coping strategies, so they were more prone to seek help for their SB problems. However, this hypothesis is still not explaining why the proneness for some coping strategies was significantly more expressed in the NSB&MSB subgroup – as these participants were taking part in the study despite more “maladaptive” characteristics of their coping mechanisms.

One crucial fact to be considered when interpreting existing data is that the majority of available studies are based exclusively on self-assessment observation questionnaires on sleep bruxism (9–11, 13). A significant difference between self-reported and PSG assessments of sleep quality in patients with depressive symptoms and TMD has been reported (36). Similar differences should be considered when interpreting SB based solely on self-reported studies. Self-assessment approaches can only determine a possible SB diagnosis. PSG evaluation is considered to be a gold standard required for the “definite diagnosis” of SB (9–11, 17). The present study aimed to determine the correlations between selected psychological risk factors (such as personality traits, coping strategies, and depressive and anxiety symptoms) and SB based on PSG examinations according to the international consensus (by Lobbezoo et al.) (1).

Available data also show that a decrease in both positive and depressive feelings is not exclusively associated with SB but is also associated with arousal and other malfunctions (10), suggesting low specificity of these symptoms regarding SB. SB coexistence with sleep-related disorders and many consequences for oral and overall health have been previously reported (2–5).

Some investigators proposed an emotional hypothesis to explain SB regulation (37). Depression and negative affect, measured as both inward anger and aggression in nightmares, have been found to be greater in individuals with clinically suspected SB than in those without SB (38). Giraki (2010) emphasized that individuals with suspected high SB activity seemed to feel more stressed in their daily lives and at work (11). This disagrees with our findings, as we found strong positive correlations in the case of OBC scores and depressive and anxiety symptoms but not in the case of SB activity. A study by Miletić et al. showed that patients with sleep bruxism have higher levels of salivary cortisol (a stress marker) (39). However, stress biomarkers like salivary cortisol levels were not assessed in our study. Arecent meta-analysis of 10 studies published by Polman et al. identified that individuals with probable SB had higher levels of some self-reported stress symptoms and biomarkers, emphasizing that the quality of evidence was very low, and caution should be exercised in interpreting these results (40). The nature of correlations of anxiety and depressive symptoms and SB activity remains unclear, though our study that included participants with definite diagnosis of SB sheds a light on a possible new perspective for future studies. Additionally, it confirms the importance of PSG evaluation in case of SB suspicion.

A recent study by Saczuk et al., based on a portable screening instrumental approach for SB (EMG portable screening device), showed a relationship between perceived stress (measured by PSS-10) and sleep bruxism and suggested that maladaptive coping strategies (by Brief-COPE) were chosen more frequently in the SB group than in controls (41). In a PSG-based study by Smardz et al., SB did not significantly correlate with self-reported perceived stress (12). Negative coping strategies were also suggested in a study that assessed SB with the Bruxcore Bruxism Monitoring Device, but there was no masticatory muscle action monitoring; therefore, SB could be mistaken for other TMDs (11). Some cited results (11, 41) should be interpreted with caution because of the heterogeneity of the study groups, including smokers and patients with obstructive sleep apnea syndrome, which may have significantly affected the intensity and possible background of SB (16, 42).

Recent data on isolation during the COVID-19 pandemic emphasized that maladaptive coping strategies were chosen by participants experiencing high levels of stress, purely coexisting with both self-reported TMD and bruxism symptoms (43). Moreover, self-reported coping strategies that enhanced stress in another study did not appear to be associated with probable sleep bruxism (13). This indicates that the group of sleep bruxers in many studies is heterogeneous, and SB intensity may be associated with different coping strategies. In our study, in terms of coping mechanisms, the PSG-confirmed homogenous SSB group was less likely to use self-distraction, denial, venting, behavioral disengagement, self-blame, and turning to religion than the no SB/mild SB participants. Additionally, in the final model analysis of our study, behavioral disengagement strategy was a protective factor for SSB (measured by BEI), which clearly showed no maladaptive coping strategies in definite PSG-diagnosed severe sleep bruxers. This is in agreement with the Manfredini report, one of the few available studies based on an instrumental approach for SB diagnosis (EMG portable screening device), in bruxism activity higher-scoring healthy participants; there were significant correlations between the prevalence of SB and social support coping strategies (44). These findings support our present observation that negative strategies are less likely to be used in SSB patients. Interestingly, no coping strategies were seemingly preferred by SSB participants in our study. This observation requires further research, as it should be explained if SB might be a form of somatization preferred by patients over the coping strategies used during the day. The participants were unaware of this unconscious and nocturnal mechanism; therefore, they might not have been reported in the questionnaires. Some authors have suggested that SB may be a stress-coping strategy and could be recognized as a valid systemic prophylaxis for stress (45). The term “somatization” was initially introduced in psychoanalysis and it pointed out connections of such symptoms to dissociation or conversion mechanisms. Somatization has also been attributed to specific personality profiles and categories of stress or dysfunctional behavioral patterns (A, C, or D). Much more complex mechanisms of action have been proposed based on neuroimaging and electrophysiological studies. The potential mechanisms studied include immunological system regulation, vegetative (autonomic nervous system), hypothalamic-pituitary axis malfunctions, mitochondrial function abnormalities, perception and processing of bodily signals, central sensitization, and psychological adaptation. Finally, psychological mechanisms concerning predictive processes within cognitive homeostasis dysregulation in the human brain and the free energy flow principle in the brain have also been proposed as mechanisms underlying somatization (46). Interestingly, in our study, the phasic activity correlated negatively with the BAI score. It would suggest, at least in the group of SSB participants with predominant phasic activity, that a possible psychological or functional (“somatization”) mechanism could underlie the SB phenomenon. Tonic activity seems to be more likely connected to respiratory events in some studies (47), while according to other studies, such events seem to be more related to phasic activity (48). In our study tonic activity was not widely present, though it remains unclear if it was due to the exclusion of participants with OSA or it was a coincident, or perhaps both. Again, further research on non-OSA participants with SB would be required to explain the differences in different mechanisms underlying phasic and tonic activity.

It has been suggested that subjects with higher levels of anxiety, depression, and increased disclusion time may have a greater predilection for bruxism (49). A positive relationship was found between self-reported AB and anxiety symptoms, whereas a similar relationship was not observed for SB (50). In another study, clinically probable SB was associated with somatic anxiety but not cognitive anxiety, depression, or anger (32). Another meta-analysis by Polmann et al. concentrating on the SB connection with anxiety showed that the data were controversial, as no study with a definite assessment of SB was identified. No association with SB was observed in three studies that investigated generic levels of anxiety, whereas two other studies found that some symptoms of the anxiety disorder spectrum (measured using the State-Trait Anxiety Inventory (STAI) and panic-agoraphobic spectra evaluation (PAS-SR) questionnaire) may be associated with probable SB (51).

For the Big Five personality traits model it is necessary to notice that many studies on SB have not assessed personality traits (11, 13, 41, 43). Self-reported mixed bruxism and bruxism have been reported to be mainly associated with personality traits of neuroticism and extraversion, which partially supports our observation (52, 53). Alternatively, in a Japanese study, intra-aggression and lack of adequate self-assertiveness were more prevalent in SB than in non-SB participants, but not in a PSG-controlled study (54). Factors such as depression, hypomania, and suppressed aggression have also been found to be common in patients with sleep bruxism (39). Some data also support the hypothesis that anxiety, as a personality trait, might be related to SB, specifically the duration of sleep-time masticatory muscle activity (44, 55). Provided data are debatable, which underlines the more complex relationship between SB and psychological features and requires further examination of big homogenous groups based on gold standard methodology to gain good quality of evidence. Contrary to mentioned articles, in our study there were no significant predictors of SB found among the Big Five personality traits, though both studies groups differed in the expression of most of these traits.

Regarding the OBC scores in our study, in the regression model, emotional stability was a relatively moderate protective (negative) factor, and the self-blame strategy was a relatively strong risk (positive) factor for increased OBC scores. This suggests that oral parafunctional behaviors have a psychogenic background, occurring more often in cases of less emotionally stable personalities and in people who are eager to use self-blame coping strategies. Several studies have pointed out that in adolescent populations, self-blame strategies are possible “mediators” or risk factors of self-harm acts (56, 57). Emotional intelligence has been mentioned as a protective factor against self-harm in a study on an adolescent population (58). Still, these findings need to be compared carefully to our findings, as the present study enrolled only adult participants and used different tools for the assessment of coping strategies, and emotional intelligence is not understood in the same way as the emotional stability personality trait in the Big Five Model. Further research could explore whether oral parafunctional behaviors can be perceived as a phenotype (or a substitute) for self-harm (or other types of emotion regulation behaviors). Moreover, to our knowledge, self-harm in adult sleep or awake bruxers has not been discussed or explored in the literature.

### 4.1 Limitations

This study has several limitations. First, PSG evaluation was performed without night adaptation, which may have affected some of the results. However, a recent study focused on the possible first-night effect on SB and showed that for mild and SSB, a single-night evaluation might be sufficient, while the low RMMA activity group could benefit from second-night verification (59). Second, the IPIP-BFM-20 and Mini-COPE are self-assessment tools; therefore, there is always a lack of verification with a clinician-applied tool. However, both tools are widely used in clinical studies, as described in the methodology section. Similarly, the BAI and BDI are self-assessment questionnaires that are insufficient to establish a clinical diagnosis of a depressive or anxiety disorder. However, the goal of their study was to measure the intensity of symptoms and not to establish a clinical diagnosis. As discussed in the methodology section, both tools are widely used in this context. Third, though the gender structure of both subgroups are statistically comparable, the quantity of female participants is bigger, what could possibly affect the results. Finally, due to the relatively low R2 values in the regression models presented in the study, the results in terms of possible protective and risk factors should be interpreted with great caution and require further verification in larger studies that also assess other known risk factors, such as biological ones. Similarly, most of the observed correlations were of weak to moderate strength; therefore, they should be interpreted with caution. Observations described in this paper are correlational, thus they should not be interpreted as causal. However, as initial findings they encourage further research in order to assess any possible causal relationships of the described aspects of SB, personality traits and coping strategies. Last, the participants were enrolled to the study with a suspicion of SB based on a dental examination. Although in some of the cases the PSG evaluation excluded the SB (as a gold standard diagnostic method), it is debatable if the participants without SB can be considered as “healthy subjects”.

## 5 Conclusions

SSB was correlated with less severe anxiety and depressive symptoms, whereas extraversion, emotional stability, and intellectual traits were more pronounced in SSB. There was no difference in the intensity of daytime oral parafunctional behaviors between severe sleep bruxers and non-bruxers. SSB seems to predispose individuals to less use of the following coping strategies: turning to religion, self-distraction, denial, venting, behavioral disengagement, and self-blame (compared to no or mild SB). No coping strategies were preferred by participants with SSB. Self-blame strategy turned out to be a possible protective factor for tonic activity, while venting was a potentially weak protective factor for mixed activity. Phasic activity seemed to be negatively correlated with anxiety symptom severity, whereas tonic and mixed phenotypes were not. The SB intensity (measured with BEI) was found to be a possible protective factor against anxiety symptoms. These observations require further studies, as it should be explained if SB (especially phasic activity) might be a form of a functional (“psychosomatic”) disorder. Behavioral disengagement strategy was possibly a relatively weak protective factor against SB intensity, while other coping strategies were found to be insignificant factors, though some of these strategies (turning to religion, denial, venting and self-blame) correlated with SB intensity. Emotional stability was a moderate protective factor, and self-blame strategy was a strong risk factor for increased oral parafunctional behavior intensity. This suggests that oral parafunctional behaviors might have a psychogenic background, occurring more often in cases of less emotionally stable personalities and in people who are eager to use self-blame coping strategies. Further research should explore whether such behaviors can be perceived as a phenotype (or substitute) for self-harm. In general, more further research should focus on verification of these (mostly correlational) observations to determine their possible causal nature.

## Data availability statement

The original contributions presented in the study are included in the article/supplementary material. Further inquiries can be directed to the corresponding author.

## Ethics statement

The studies involving humans were approved by Bioethical Committee at Wroclaw Medical University. The studies were conducted in accordance with the local legislation and institutional requirements. The participants provided their written informed consent to participate in this study.

## Author contributions

TW: Conceptualization, Data curation, Formal analysis, Investigation, Methodology, Software, Writing – original draft, Writing – review & editing. AJ: Data curation, Writing – original draft. SO: Conceptualization, Data curation, Investigation, Methodology, Writing – review & editing. MW: Conceptualization, Investigation, Methodology, Project administration, Resources, Supervision, Writing – review & editing. MM-Z: Data curation, Formal analysis, Investigation, Writing – review & editing. GM: Funding acquisition, Resources, Supervision, Writing – review & editing. JR: Supervision, Writing – review & editing. JS: Data curation, Investigation, Resources, Writing – review & editing. AW: Data curation, Resources, Writing – review & editing. HM: Conceptualization, Data curation, Funding acquisition, Investigation, Methodology, Project administration, Resources, Supervision, Writing – review & editing.
